# Gut microbiota and associated metabolites: key players in high-fat diet-induced chronic diseases

**DOI:** 10.1080/19490976.2025.2494703

**Published:** 2025-04-22

**Authors:** Wei Du, Zhen-Ping Zou, Bang-Ce Ye, Ying Zhou

**Affiliations:** Laboratory of Biosystems and Microanalysis, State Key Laboratory of Bioreactor Engineering, East China University of Science and Technology, Shanghai, China

**Keywords:** High-fat diets, chronic diseases, gut microbiota, metabolites

## Abstract

Excessive intake of dietary fats is strongly associated with an increased risk of various chronic diseases, such as obesity, diabetes, hepatic metabolic disorders, cardiovascular disease, chronic intestinal inflammation, and certain cancers. A significant portion of the adverse effects of high-fat diet on disease risk is mediated through modifications in the gut microbiota. Specifically, high-fat diets are linked to reduced microbial diversity, an overgrowth of gram-negative bacteria, an elevated *Firmicutes*-to-*Bacteroidetes* ratio, and alterations at various taxonomic levels. These microbial alterations influence the intestinal metabolism of small molecules, which subsequently increases intestinal permeability, exacerbates inflammatory responses, disrupts metabolic functions, and impairs signal transduction pathways in the host. Consequently, diet-induced changes in the gut microbiota play a crucial role in the initiation and progression of chronic diseases. This review explores the relationship between high-fat diets and gut microbiota, highlighting their roles and underlying mechanisms in the development of chronic metabolic diseases. Additionally, we propose probiotic interventions may serve as a promising adjunctive therapy to counteract the negative effects of high-fat diet-induced alterations in gut microbiota composition.

## Introduction

High-fat diets (HFD) are gaining popularity in contemporary society.^1^ The health risks associated with the overconsumption of fat are becoming more apparent. Research demonstrates that sustained adherence to an HFD substantially elevates the risk of developing a range of chronic conditions, including diabetes, obesity, cardiovascular disease, hepatic metabolic disorders, and certain cancers. These health issues not only aggravate individual well-being but also present a formidable challenge to public health systems.^2^ As a result, investigating the factors that contribute to the elevated risk of diseases associated with HFD, along with identifying strategies to mitigate the detrimental health effects of such dietary patterns, has emerged as a critical focus within the field of public health.

Recent epidemiological and omics-based studies underscore the significant role of microbial communities in mediating the environmental influences on human health and disease susceptibility.^3^ The human gut harbors a diverse array of microbes that are shaped by various factors, including mode of delivery, diet, lifestyle choices, medication use, and host genetic predisposition.^3^ The gut microbiome plays a pivotal role in shaping host immunity, facilitating food digestion, regulating gut endocrine function and neural signaling pathways, modulating drug efficacy and metabolism processes, detoxification mechanisms, as well as synthesizing various bioactive compounds impacting the host’s overall physiology.^3^ Numerous studies have demonstrated the essential involvement of gut microbiota in the pathogenesis of HFD-related chronic diseases.^4–8^

The composition of the gut microbiota is profoundly influenced by the quantity of dietary fat. HFD has been associated with a reduction in the diversity of the gut microbiota,^9^ as well as an increase in the translocation of lipopolysaccharide (LPS),^10^ heightened intestinal permeability,^11^ elevated inflammation,^12^ and disrupted immune system function.^13^ Furthermore, HFD-induced alterations in the intestinal microbiota lead to shifts in the metabolic activity of gut microbes, impairing host metabolic processes such as glucose^14^ and lipid metabolism,^15^ signal transduction,^16^ and endotoxin accumulation.^17^ These disruptions contribute to an increased risk of chronic metabolic disorders associated with HFD. Therefore, there has been growing interest in elucidating the role of dietary fat-induced changes in the gut microbiota, particularly in relation to obesity and associated chronic metabolic diseases. This review aims to investigate the relationship between HFD and gut microbiota, focusing on their roles and underlying mechanisms in chronic metabolic diseases. Additionally, we address crucial considerations and limitations in examining the impact of microbiota on host pathology. Finally, we propose probiotic therapy as a potential adjunctive strategy to mitigate the adverse effects of HFD-induced modification in gut microbiota composition.

## Effects of HFD on gut microbiota

Research on the human microbiome highlights that important environmental influences on humans may arise from changes in the microbial communities within the gastrointestinal tract. These microorganisms, collectively known as the “gut microbiota,” encompass a diverse array of interacting bacteria, archaea, phages, eukaryotic viruses, and fungi. They coexist harmoniously within the human gut and establish predominantly symbiotic or mutualistic relationships with their hosts. The gut microbiota represents a dynamic system that is influenced by various factors including environment, diet, lifestyle choices, medications, and host genetics. Intestinal microorganisms play pivotal roles in modulating host intestinal immunity, facilitating nutrient digestion and absorption processes, influencing drug efficacy and intestinal metabolic responses while exerting systemic effects via intricate communication pathways such as the gut-brain axis, gut-liver axis, and gut-heart axis.^3,18–20^

### Dietary modulation of gut microbiota

Defining a taxonomically precise “healthy gut microbiome” is a crucial aspect of microbiome research. However, such definition remains elusive for the human gut microbiome. The variability among host individuals renders each person’s gut microbiome unique, which further complicates the establishment of a standardized definition for a “healthy gut microbiome”. The perturbation of the gut microbiome in disease states can be conceptualized as an imbalance within the ecosystem of a healthy gut microbiome. The human microbiome primarily comprises five bacteria (*Firmicutes, Bacteroidetes, Proteobacteria, Actinobacteria*, and *Verrucobacteria*) and one archaeon, with their relative distribution being unique to each individual host. Generally, anaerobic bacteria with restrictive metabolism dominate over facultative anaerobic bacteria.^21^ Despite substantial inter-individual variations, high-class diversity, abundant microbial gene richness, and stable core microbiota are characteristic features of a healthy gut microbiota.^22^

The diet plays a crucial role in shaping the composition and coordinating host-microbe interactions in the gut microbiome. The composition of the gut microbiome is sensitive to dietary components and reacts differently to diet in different environmental backgrounds. Dietary components can directly promote or inhibit the growth of specific gut microbes. Gut microbes with the ability to obtain energy from specific dietary components have a competitive advantage, allowing them to grow better than those that are not good at this function. In addition, dietary vitamin A deficiency leads to excessive growth of *Bacteroides vulgatus* in mice, which may be due to the inhibitory effect of vitamin A on bacteria.^23^ Vitamin D is crucial for combating intestinal pathogens and maintaining the survival of beneficial symbiotic organisms.^24^ Dietary *lactobacilli*, *candida*, and *penicillium*, among others, can also be passively transferred and colonized in the host gut microbiome through diet.^25^ However, the extent of colonization is also contingent upon the preexisting microbe, primarily due to differences in the resistances and receptiveness of unique gut microbiome compositions to foreign bacterial colonization.^26^ Dietary components may disrupt the protective function of the gut barrier indirectly, leading to dysbiosis, such as HFD and low-fiber diet being believed to disrupt the barrier function of mice, ultimately leading to the promotion of mild inflammation and metabolic syndrome mediated by dysbiosis.^16,27,28^ There are significant differences in gut microbiota composition among individuals, populations, and dietary patterns, reflecting complex interactions of host genetic background, lifestyle, environmental exposure, and long-term dietary habits. Different individuals may respond very differently to the same diet, depending on the initial composition and metabolic capacity of their gut microbiota. For example, certain populations may have unique microbiome structures due to their traditional dietary patterns, such as high-fiber or fermented foods, that show greater adaptability and stability in responding to specific dietary challenges. In addition, regional and cultural differences also lead to distinct characteristics in the gut microbiota of different populations, such as differences in microbiota composition between Western high-fat, low-fiber dietary patterns and traditional high-diversity, high-fiber dietary patterns. Understanding microbiome differences among individuals, populations, and dietary patterns is therefore critical to developing personalized nutrition intervention strategies and promoting gut health.

### HFD-induced gut microbiota dysbiosis

**Figure 1.**
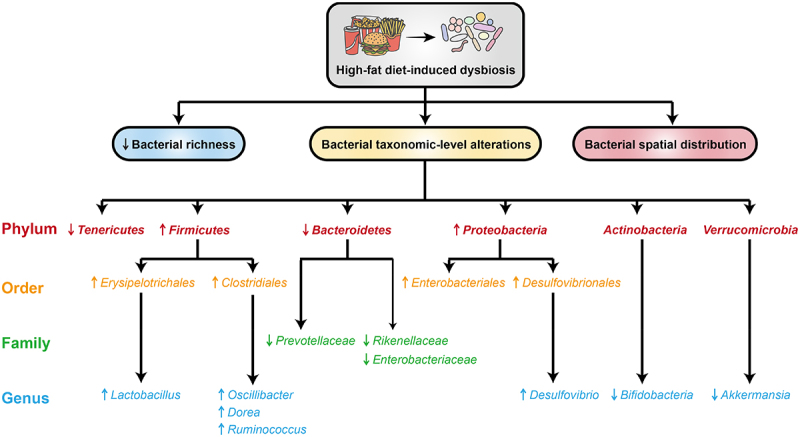
Effects of HFD on gut microbiota dysbiosis.

Although the composition of the gut microbiome varies among individuals, there is substantial evidence linking dietary fat to composition of microbiome. Despite host genotypes can shape the gut microbiota by modulating immune system, their impact is frequently eclipsed by the predominant influence of diet.^29^ Gut microbiota disorders related to obesity are directly associated with HFD, which promotes the development of a similar microbiota as observed in obese male mice.^16,30,31^ The microbiota of HFD mice exhibited an overall reduction in microbial diversity and alterations in the relative abundance of various bacterial taxa (Figure 1).^32,33^ Moreover, transplantation of fecal bacteria from mice on a normal diet (ND) to HFD mice can effectively diminish their food utilization efficiency and significantly enhance their metabolic function.^34^

Dietary fats have been demonstrated to induce alterations in the composition of the gut microbiota, notably through decreasing the abundance of the *Bacteroidetes* phyla while increasing the abundance of *Firmicutes* and *Proteobacteria* phyla.^35,36^ The *Firmicutes/Bacteroidetes* ratio is commonly employed as a key indicator of microbial shifts, serving as a marker for changes in intestinal microbiota composition.^37^ In both animal and human studies, dietary fat typically results in an elevated *Firmicutes/Bacteroidetes* ratio.^16,38^ This shift is primarily attributed to an increased abundance of specific genera within the Firmicutes phylum, including *Oscillibacter*, *Dorea*, *Ruminococcus*, *Lachnospira*, and *Lactobacillus*.^16,39,40^ Furthermore, dietary fat consumption has been linked to decreased levels of *Prevotellaceae* and *Rikenellaceae* families within the Bacteroides phylum.^16,41,42^ Additionally, dietary fat promotes the proliferation of *Proteobacteria*,^43^ a group containing pro-inflammatory, Gram-negative bacteria such as those from the *Enterobacteriales* order, which possess LPS.^44^ Chronic high-fat diets have also been linked to a significant decrease in the abundance of *Tenericutes* in the murine intestinal microbiota.^45^ Moreover, the abundance of the genus *Akkermansia* has been reported to exhibit a negative correlation with body fat percentage in HFD mice,^46^ a similar association being observed in obese or overweight human individuals, particularly with regard to *Akkermansia muciniphila*.^47^ HFD-induced dysbiosis is often linked to a decrease in *Bifidobacteria (Actinomycetes* phyla), which is inversely correlated with intestinal barrier integrity.^48^ However, this relationship is not universally supported across all studies, with some evidence contradicting the anticipated positive correlation. The discrepancies observed in the literature can be attributed to several factors, including microbial adaptability to dietary changes, intervention duration, individual genetic variations, environmental exposure differences, and heterogeneity in sample collection and data analysis methodologies. For instance, research has demonstrated that the gut microbiome’s response to dietary interventions varies significantly among individuals, potentially due to differences in baseline microbiome composition, host metabolic status, and lifestyle factors.^25,49^ Moreover, the duration of the intervention may influence the pattern of microbiome changes, with short-term interventions possibly failing to capture long-term microbiome dynamics.^50,51^ The highly individualized and dynamic nature of the gut microbiome complicates the establishment of universal biomarkers. Additionally, interactions between the microbiome and the host involve multilayered regulatory networks, including metabolites, immune signaling, and neuroendocrine pathways, which remain incompletely understood. As a result, single-dimensional microbiome analyses often fail to capture the full spectrum of their association with disease. To address these challenges, integrating complementary omics approaches and advancing next-generation sequencing technologies are essential. Multi-omics integration, such as combining metagenomics and metabolomics, can provide a more comprehensive understanding of microbiome-host interactions, enabling precise identification of microbial metabolic pathways and disease-associated metabolites. Furthermore, artificial intelligence (AI) and machine learning algorithms offer powerful tools for analyzing large-scale microbiome data, identifying complex patterns, predicting disease risk, and optimizing personalized interventions. For example, deep learning models can extract key features from extensive datasets to develop predictive models for early diagnosis and prognosis. In conclusion, integrating multi-omics approaches, advancing sequencing technologies, and leveraging computational tools will enable more accurate and insightful microbiome analysis in the future.

In a mouse model, HFD was found to induce a reorganization in the spatial arrangement of the microbiota within the intestines. Specifically, there was an increased density of microbiota colonizing the intervillous region of the ileum, accompanied by significant alterations in the composition of the colonic microbiota.^52^ Moreover, it was observed that antimicrobial peptide expression decreased in regions with higher bacterial density within the ileum. Notably, stimulation of PPAR restored proper spatial distribution patterns of intestinal flora; conversely, PPAR-deficient mice exhibited enhanced colonization within the ileum.^52^ These findings provide evidence for how HFD can impact both the distribution and physiological aspects of microflora within the ileum through regulation via the PPAR-γ pathway.

## HFD-Induced gut microbiota and associated metabolic alterations in chronic diseases

Gut microbial metabolites serve as crucial mediators in maintaining health and influencing disease progression, facilitating key interactions between the host and its microbiota. The homeostasis of metabolites directly impacts host metabolism, immunity, and barrier function. Short-chain fatty acids (SCFAs), produced through the microbial fermentation of dietary fibers such as acetic acid, propionic acid, and butyric acid, serve as key metabolites in intestinal energy metabolism. Butyric acid is a primary energy source for colon epithelial cells and plays a crucial role in maintaining intestinal barrier integrity by upregulating tight junction protein expression.^53^ Furthermore, it promotes the differentiation of regulatory T cells (Tregs) and mitigates excessive inflammatory responses.^54,55^ LPS constitutes a major component of the outer membrane of Gram-negative bacteria. Upon translocation across the intestinal barrier into the bloodstream, LPS binds to Toll-like receptor 4 (TLR4) on host immune cells, thereby activating the downstream NF-κB signaling pathway. This activation promotes the release of inflammatory cytokines and has been implicated in both metabolic syndrome and inflammation.^56,57^ A reduction in the abundance of SCFAs-producing bacteria and an increase in LPS-producing opportunistic pathogens, such as *Klebsiella pneumoniae*, are correlated with disease activity and recurrence in chronic spontaneous urticaria (CSU). Zhu et al. substantiated the role of gut microbiota in CSU pathogenesis through fecal microbiota transplantation (FMT) experiments.^58^ The biological effects of amino acid metabolites exhibit dual characteristics. Tryptophan is metabolized by gut microorganisms into indole derivatives which enhances blood-milk and intestinal barrier function and exerts anti-inflammatory effects through activation of the aryl hydrocarbon receptor (AhR).^59,60^ Conversely, excessive accumulation of branched-chain amino acids (BCAAs) has been shown to potentially induce insulin resistance.^61^ In conclusion, gut microbiota-derived metabolites from the form an intricate network of microbial-host interactions by modulating immune responses, metabolic pathways, and barrier functions. The equilibrium of these metabolites can serve as potential disease biomarkers and provide a theoretical foundation for developing intervention strategies that target the microbiome.

**Figure 2.**
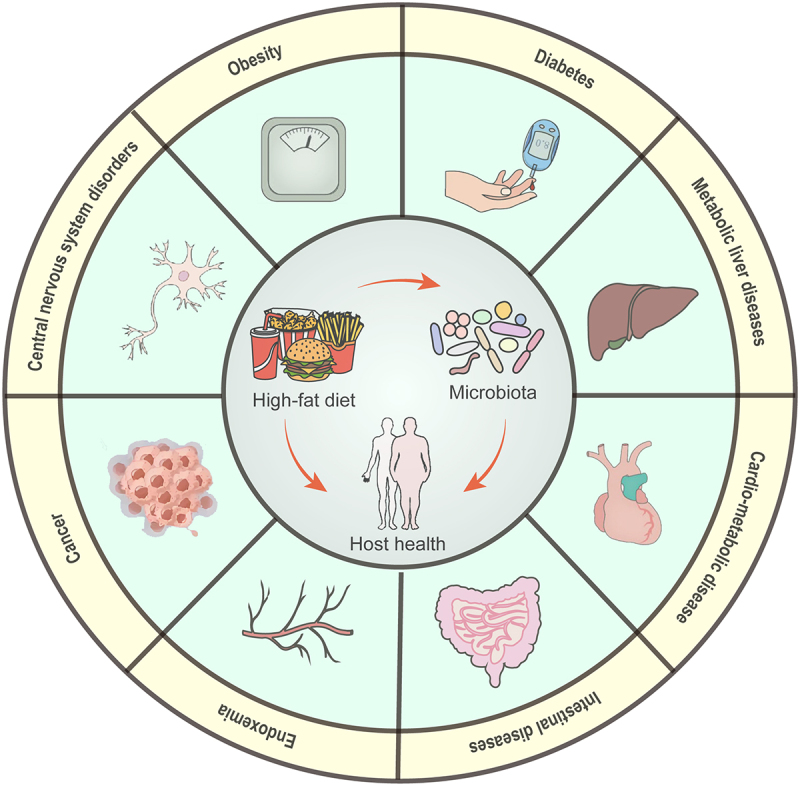
HFD-induced gut microbiota disturbances are associated with common chronic metabolic diseases.

**Figure 3.**
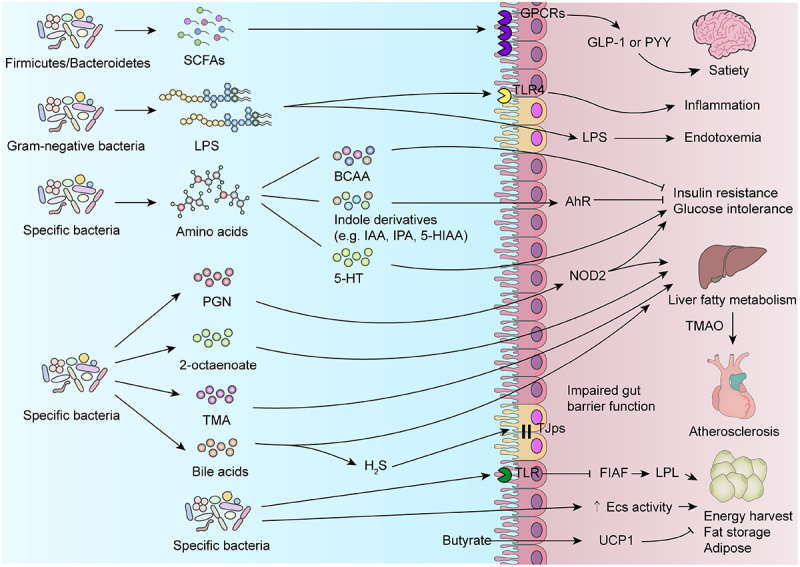
Microbial metabolites associated with HFD regulate host metabolism. An overview of HFD-induced changes in gut microbial metabolic responses affecting host energy homeostasis, body fat, inflammation, glucose regulation, insulin sensitivity, and hormone secretion. SCFAs: short chain fatty acids. LPS: lipopolysaccharide. BCAA: branched-chain amino acid. IAA: indole-3-acetic acid. IPA: indole-3-propionic acid. 5-HIAA: 5-hydroxyindole-3- acetic acid. 5-HT: 5-hydroxytryptamine. PGN: peptidoglycan. TMA: trimethylamine. GPCRs: G protein-coupled receptors. TLR: toll-like receptor. GLP-1: glucagon-like peptide-1. PYY: peptide YY. AhR: aryl hydrocarbon receptor. TMAO: trimethylamine oxide. TJps: tight junction proteins. FIAF: fasting-induced adipokine. Ecs: endocannabinoid systems. UCP1: uncoupling protein-1.

Long-term consumption of HFD increases the susceptibility to obesity, diabetes, liver metabolic disorders, cardiovascular diseases, chronic intestinal inflammation, cancer, and other related conditions (Figure 2). Recent evidence strongly suggests that the gut microbiome plays a significant role in this process. Dysregulation of gut microbes induced by HFD is a crucial factor contributing to the elevated risk of these diseases. This dysregulation may result from alterations in gut microbiota composition that promote changes in intestinal metabolic responses. These changes can lead to the production of harmful metabolites that impair host glucose and fat metabolism as well as disrupt intestinal barrier function and signal transduction pathways. Additionally, they may inhibit the synthesis of beneficial metabolites with positive regulatory effects on health, thereby facilitating disease occurrence and progression (Figure 3).

### Obesity

The prevalence of obesity is increasing globally, with variations observed across different geographical regions, ethnicities, age groups, and genders.^62^ Sedentary lifestyles and diets, combined with a widespread polygenic predisposition, are believed to be major contributors to the obesity epidemic.^63^ Moreover, mounting evidence supports the role of gut microbiome in the pathogenesis of obesity. In 2006, it was discovered that transplanting microbiota associated with obesity led to weight gain in previously lean mice.^5^ Since this finding, subsequent epidemiological studies have highlighted significant differences in the composition of gut microbiota between obese and lean individuals. At a species level, twin studies have demonstrated an association between the abundance of short-chain fatty acid-producing bacteria such as *Eubacterium ventriosum* and *Roseburia intestinalis* with obesity.^64^ A metagenomics correlation study conducted on lean and obese individuals showed a significant reduction in *Bacteroides thetaiotaomicron* abundance among obese individuals along with a negative correlation with serum glutamate concentration.^65^ Additionally, feeding mice with *Bacteroides thetaiotaomicron* prevented obesity, suggesting potential interventions targeting gut microbiota through probiotics or microbial compounds for future research purposes. A FMT study supplemented observational studies by demonstrating that transferring feces from discordant twins into germ-free mice could transfer human donor phenotype to recipient animals depending on their diet.^66^ This groundbreaking study provides a theoretical foundation for investigating gut microbiota disturbances linked to obesity.

Excessive fat intake is an environmental factor associated with obesity and metabolic disease. However, some subjects were less susceptible to weight gain and metabolic changes when similarly consuming too much energy.^67^ Suggests that in addition to the human genome, the gut microbiome is also involved in the development of obesity.^68^ The gut microbiota plays a crucial role in the production of SCFAs, which significantly influence the host’s energy harvest and storage from dietary components.^69^ SCFAs, including acetate, butyrate, and propionate, are produced by fermenting dietary fiber. They bind to g protein-coupled receptors GPR41 and GPR43,^70,71^ increasing glucagon-like peptide 1 (GLP-1) and peptide YY (PYY) expression in the gut.^72^ Studies have shown that GLP-1 and PYY can inhibit appetite^73^ in obese mice while reducing body weight and improving insulin resistance.^74^ Additionally, PYY reduces intestinal transport by inhibiting food absorption, intestinal motility, pancreatic secretion, and gastrointestinal emptying.^75^ Butyrate promotes fatty acid oxidation and thermogenesis through multiple mechanisms. In the liver and muscle, it increases the phosphorylation of peroxisome proliferator-activated receptor-γ coactivator 1α (PGC-1α) and AMP-activated protein kinase (AMPK). Additionally, in brown adipose tissue, butyrate enhances the expression of PGC-1α and mitochondrial uncoupling protein 1 (UCP-1). These effects collectively contribute to improved metabolic function.^76^ Several studies have demonstrated that HFD leads to a greater reduction in total SCFAs compared to an LFD.^77,78^ Moreover, administering a high-fat and high-sugar diet to mice led to a significant decrease in GPR43 expression compared with the control group.^77^ These studies demonstrate how SCFAs produced by gut microbes regulate host metabolism/appetite. Alterations in the gut microbiota can contribute to weight gain by augmenting energy harvested from the diet and enhancing lipid accumulation.^5,38,79^ The gut microbiota is capable of downregulating the expression of fasting-induced adipokine (FIAF) through interacting with surface molecules on intestinal endocrine cells, including toll-like receptors (TLRs).^80^ FIAF is a peptide that acts as a potent inhibitor of circulating lipoprotein lipase (LPL) activity.^81^ Inhibition of FIAF leads to increased LPL activity in adipocytes, resulting in triglyceride deposition.^79^ Moreover, it can also contribute to fat accumulation by modulating fat absorption and conversion processes. Mice lacking FIAF (-/-) exhibit elevated intestinal fat uptake and reduced fecal lipid excretion, leading to an obese phenotype.^82^ *Bacteroides thetaiotaomicron* has been shown to stimulate adipogenesis through inhibition of FIAF expression.^83^ Additionally, the endocannabinoid system (EC) is implicated in the regulation of blood lipids and glucose metabolism. Alterations in EC activity can modulate the expression of adipose tissue hormones, such as apelin, with excessive activation posing a risk for obesity.^84^ Certain gut microbiota, such as *Myxophilus*, have been found to disrupt fat metabolism by inhibiting EC-driven lipogenesis, stimulating adipocyte proliferation, and increasing lipid accumulation within adipocytes.^85,86^

### Diabetes

Evidence from large-scale epidemiological studies suggests that patients who have undergone total colectomy are at an increased risk of developing type 2 diabetes (T2D) compared to individuals without colectomy.^87^ This implies a potential involvement of the gut microbiota and the distal gut’s hormone secretion capacity in glucose regulation.T2D accounts for approximately 90% of all cases of diabetes^88^ and, similar to obesity, its incidence and prevalence are rising, affecting 5% to 15% of the adult population and making it the most common endocrine disorder.^89^ The early pathophysiological features of overweight or obesity primarily involve insulin resistance in skeletal muscle, liver, and adipose tissue, along with compensatory increases in insulin synthesis and secretion. In cases where persistent insulin resistance occurs, there is a decrease in insulin biosynthesis followed by elevated hyperglycemia.^90,91^ However, since T2D patients require treatment with multiple drugs targeting high blood sugar levels, this multi-drug regimen also affects their gut microbiota differently. Consequently, recent epidemiological studies have aimed to elucidate the connection between gut microbiota and T2D during the prediabetes phase. Prediabetic individuals exhibit elevated blood sugar values within the non-diabetic range but at levels that increase their risk of developing significant T2D.^6^ In these non-medicated prediabetic patients, alterations were observed in their gut microbiota composition, characterized by a reduction in butyrate-producing taxa and an increase in bacteria with pro-inflammatory potential.^6,92^ Furthermore, gestational diabetes mellitus (GDM), which affects 7–10% of pregnancies in the third trimester, represents another prediabetic state.^93^ Compared to pregnant women with normal blood glucose levels, those with GDM exhibit disrupted gut microbiota composition. Notably, during and after pregnancy, the gut microbiota of individuals with GDM resembles that of non-pregnant individuals with T2D who have abnormal microbiota.^94^

A representative animal model of diabetes is the mouse model fed HFD, which exhibits high reproducibility and similarity to human disease pathogenesis. Consequently, it is extensively employed in investigating the phenotype and pathology of diabetes. There is an increasing focus on exploring the association between HFD-induced intestinal microbiota imbalance and diabetes. Despite sharing the same background and nutritional status, diabetic or non-diabetic mice fed HFD display distinct gut microbiome profiles that are closely related to metabolic phenotypes.^95^ These results are consistent in humans, as both insulin-resistant and insulin-sensitive obese individuals display distinct microbiome profiles, even though they have comparable levels of insulin activity.^96^ A study investigating antibiotic therapy provided evidence supporting the involvement of gut microbes in HFD-induced diabetes, as evidenced by significantly decreased fasting blood glucose and insulin levels, along with improved glucose and insulin tolerance in mice treated with antibiotics.^97^ This effect may be attributed to the reduction of tryptophan metabolite 5-HT content in the host, which is regulated by gut microbes.^98^ Both antibiotics and 5-HT synthesis inhibitors significantly improved glucose tolerance in mice. However, the combination of these two treatments did not result in an additive effect on glucose tolerance, suggesting that both interventions target the same glucose metabolic pathway. Specifically, gut microbiota can directly influence the production of 5-HT by enterochromaffin cells, thereby regulating glucose homeostasis via the 5-HT pathway. The inefficiency of the gut microbiota in producing ligands for tryptophan-derived aromatic hydrocarbon receptors may also be implicated in the pathogenesis of diabetes. Both HFD mice and diabetic patients exhibited a significant reduction in AhR expression,^99^ which can be attributed to decreased production of ligands by the gut microbes through the indole metabolic pathway, including indole, indole acetic acid, tryptamine, and 5-hydroxyindole acetic acid.^16,99–101^ Treatment with FIZC (an AhR activator) rescued AhR impairment in HFD mice and ameliorated metabolic dysfunction.^99^ This effect could potentially be attributed to indole metabolites produced by the intestinal flora that promote GLP-1 secretion^100^ and insulin signaling via AhR.^16^ In addition to tryptophan, the levels of BCAAs such as L-leucine, L-valine, and L-isoleucine also exhibited significant changes following consumption of HFD.^102^ This observation aligns with the changes seen in the serum of T2D patients, characterized by elevated levels of BCAAs in individuals with reduced insulin function.^61^ This phenomenon is linked to specific alterations in the composition of the gut microbiota, notably an increase in BCAA-biosynthesizing bacteria such as *Prevotella copri* and *Bacteroides vulgatus*. And feeding these bacteria leads to impaired insulin sensitivity and glucose tolerance,^61^ highlighting the role of BCAAs and their metabolizing bacteria in the development of insulin resistance. In addition, a recent study has elucidated the involvement of NOD2 in HFD HFD-induced diabetes, as impaired peptidoglycan perception by NOD2 promotes dysbiosis and insulin resistance.^103^ Furthermore, there was an increase in bacteria involved in synthesizing SCFAs, while bacteria responsible for converting SCFAs showed a decrease. Consequently, this led to the accumulation of SCFAs and subsequent development of insulin resistance in the host.^61^ It is plausible that the SCFAs receptor GPR43 also plays a role in this process. Enhanced GLP-1 secretion through regulation of GPR43 activation also improves insulin resistance in mice.^104^ Moreover, the diminished abundance of *Akkermansia muciniphila* in the gastrointestinal tract has been linked to the onset of T2D.^6^ This implies that the reduction in *Akkermansia muciniphila* caused by HFD also partially contributes to the progression of diabetes.

### Metabolic liver diseases

The prevalence of nonalcoholic fatty liver disease (NAFLD), a hepatic manifestation of metabolic syndrome resulting from HFD, ranges from 20% to 40% among the adult population in numerous countries.^105^ NAFLD represents a range of conditions, from simple fatty liver to the more severe and inflammatory variant known as nonalcoholic steatohepatitis (NASH).^106^

The disruption of the gut microbiome induced by HFD is likely to be a pivotal factor in the pathogenesis of NAFLD and NASH diseases. Studies involving human and animal fecal transplants have demonstrated that intestinal colonization by alcohol-producing *Klebsiella pneumoniae*, which is associated with an increased risk of NAFLD, accelerates the manifestation of symptoms related to this condition.^107,108^ Similar to the dysbiosis of gut microbiota induced by HFD, NASH patients exhibited significantly elevated abundance of *Enterobacteriaceae* within *Proteobacteria* phylum.^109^ Furthermore, in children with steatosis or NASH, the gut microbiota showed a higher abundance of the *Dorea* and *Ruminococcus* genera when compared to the control group.^110^ Similarly, patients with liver cirrhosis also experienced alterations of microbiota composition, characterized by increased abundances of *Proteobacteria* and *Clostridium*.^111^ This distribution in gut microbial community structure was also observed in HFD mice. The westernization of diet contributes to the deterioration of the intestinal environment. This results in elevated levels of LPS in the bloodstream, which is an endotoxin and a constituent of Gram-negative bacterial outer membrane.^112^ HFD plays a significant role in the pathogenesis of NAFLD/NASH by promoting an increased influx of LPS into the liver through leaky gut syndrome, while also inducing intrahepatic lipid accumulation and contributing to exaggerated hepatic response to LPS.^113^ Feeding mice a typical Western diet (low in choline, high in fat, and high in sugar) increased the abundance of *Blautia* bacterium in the gut of the mice, resulting in an increase in monoglycerides of 2-octagenoate. This led to symptoms similar to those seen in human NAFLD patients, including liver hypertrophy, steatosis, inflammation of liver cells, and fibrosis.^114^ Dietary fat leads to higher concentrations of intestinal oxygen and nitrates, which promotes the growth of *E. coli* and breaks down choline into trimethylamine (TMA). Choline is then converted by liver monooxygenase into trimethylamine n-oxide (TMAO),^115^ which is considered a novel biomarker for early metabolic syndrome.^116^ Patients with NAFLD have elevated blood levels of TMAO, which regulates glucose metabolism and causes adipose tissue inflammation as well as abnormal blood glucose levels by increasing serum levels of the inflammatory cytokine C-C motif chemokine 2 (CCL2).^117^ SCFAs also play a role in metabolic liver disease. Studies have shown that butyric acid mediates crosstalk between the gut and liver through the LKB1-AMPK-Insig signaling pathway. It regulates liver fat production and maintains lipid homeostasis.^118^

### Cardio-metabolic disease

The existing literature provides evidence for the impact of gut microbiota on cardiovascular disease, which is believed to be mediated through the recognition of gut microbial metabolites by host receptor systems.^119^ The progression of atherosclerosis induced by a Western diet is associated with alterations in gut microbiota functionality. Reversal of this process can be achieved by switching to a normal diet, highlighting the crucial role played by intestinal microbiota in atherosclerosis development.^120^ Individuals with Cardio-metabolic disease exhibited decreased abundances of *Bacteroides* and the anti-inflammatory *prausnitzii faecalis*.^121^ Recent investigations have revealed that ischemic heart failure is also linked to dysregulation of gut microbiota, characterized by elevated abundances of *Ruminococcus, Acinetobacter*, and *Veillonella*. Functionally, the microbiome in these patients displayed a high prevalence of genes involved in LPS and TMAO biosynthesis.^122^ Rodent experiments have demonstrated that dietary supplementation with TMAO or its precursors accelerates the progression of arteriosclerosis, promotes platelet aggregation, and enhances thrombosis.^123^ Moreover, attenuation of TMA production by gut bacteria using 3,3-dimethyl-1-butanol (a TMA lyase inhibitor) can ameliorate arteriosclerosis and thrombotic events.^124^ The gut microbiota metabolizes choline, lecithin, and L-carnitine, primarily derived from red meat, egg yolks, and dairy products, into TMA. TMA is transported to the liver via the portal vein and subsequently oxidized to TMAO by flavin monooxygenase (FMO). Mechanistically, TMAO enhances the uptake of oxidized low-density lipoprotein (ox-LDL) by upregulating the expression of macrophage scavenger receptors, including CD36 and SR-A1, thereby promoting the formation of foam cells,^125^ which serve as an early indicator of atherosclerosis. Meanwhile, TMAO can downregulate the expression of key bile acid synthesis enzymes, including CYP7A1 and CYP27A1, as well as reduce the expression of intestinal cholesterol transporters like Niemann-Pick C1-like 1 (NPC1L1) in the liver.^126^ The inhibition of bile acid synthesis and transport leads to a diminished bile acid pool, consequently impairing reverse cholesterol transport (RCT) and ultimately resulting in cholesterol accumulation within the vascular wall, thereby promoting atherosclerosis. Moreover, TMAO potentiates platelet reactivity to agonists like collagen and ADP, thereby augmenting platelet aggregation and elevating the risk of thrombosis.^123^ TMAO also can activate the MAPK and NF-κB signaling pathways in vascular smooth muscle cells and endothelial cells, resulting in the upregulation of inflammatory gene expression and increased adhesion of white blood cells to the endothelium.^127^ Similarly, administration of antibiotics to LDLR-deficient mice effectively suppressed the gut-derived release of LPS, thereby reversing the atherosclerosis-promoting effects induced by consumption of a Western diet.^128^ An increase in *Clostridium* resulting from HFD may contribute to the development of cardio-metabolic diseases by modulating amino acid metabolism in the gut. The intestinal symbiotic bacterium *Clostridium sporogenes* metabolizes phenylalanine into phenylacetic acid (PAA) and phenylpropionic acid (PPA).^129^ In the human body, PAA combines with glutamine (Gln) under the action of liver enzymes to form phenylacetylglutamine (PAGln). PAA exhibits a positive correlation with thrombotic events in human plasma. Furthermore, it has been confirmed that PAGln can promote thrombosis formation through activation of Adrenergic Receptors (ADRs).^130^

### Intestinal diseases

The gut serves as the primary interface between an individual and their external environment, playing a crucial role in both nutrient absorption and safeguarding against ingested toxins and microorganisms. Comprising mucus layers, intestinal epithelial cells (IECs), tight junctions (TJs), immune cells, and the gut microbiome, the intricate intestinal barrier system is susceptible to various external factors including diet.

Dietary fats have been implicated in compromising this barrier integrity, triggering inflammatory responses, disrupting the structural integrity of the intestine. This predisposes individuals to a spectrum of gastrointestinal disorders such as inflammatory bowel disease, celiac disease, and irritable bowel syndrome. Studies have demonstrated that HFD feeding significantly increases the abundance of LPS-containing bacteria in the gut microbiota.^131^ Mice fed HFD experience greater intestinal barrier permeability and reduced expression of tight junction proteins compared to the control group. This is linked with substantially higher levels of the LPS receptor (CD14) and increased colonic mRNA expression of TLR4.^132^ TLR4 is part of the pattern recognition receptor family, and its activation can induce the release of pro-inflammatory cytokines while increasing intestinal permeability.^133^ LPS directly regulate TJ proteins and enhance the permeability of Caco-2 monolayer through TLR4-CD14-mediated nuclear factor kappa B (NF-κB) activation, thereby altering intestinal barrier integrity.^134^ Moreover, LPS directly induces rapid shedding of intestinal epithelial cells without compensatory TJ resealing mechanisms.^135^ Lastly, LPS may promote mitochondrial autophagy and global mitochondrial dysfunction, leading to increased intestinal permeability.^136^ HFD has been linked to increased levels of *Desulfovibrio* species, especially *Bilophila wadsworthia*. This bacterium generates genotoxic hydrogen sulfide (H_2_S) gas, which contributes to the underdevelopment and increased permeability of IECs.^137^ *B. wadsworthia* is capable of producing hydrogen sulfide, which hampers the oxidation process of butyrate and disrupts the energy balance within intestinal cells. Consequently, this leads to damage in intestinal cell structure, resulting in hypoplasia and hyperpermeability of the intestinal epithelial cells. Ultimately, these cascading effects culminate in intestinal leakage and inflammation.^138^ Furthermore, *Bilophila wadsworthia* employs sulfur obtained from taurine cholic acid (TCA) as a reducing agent in its electron transport chain to support its survival and growth within the gastrointestinal tract.^139^ In addition, HFD augments the nutritional benefits of *Oscillibacter spp*., which is directly linked to the suppression of IEC expression of TJ proteins and subsequent dysfunction in the intestinal barrier. Conversely, HFD leads to a reduction in *Bifidobacterium and Lactobacillus* populations, which are associated with improved integrity of the intestinal barrier.^140,141^ The mechanisms through which these microorganisms exert their effects on gut health remain unknown. However, they appear to stimulate gene expression of TJP within the intestine.^142–144^ Similarly, *Akkermansia muciniphila* not only induces gene expression of *Tjp1* and *Ocln* but also mitigates HFD-induced thinning of the small intestinal mucus layer (SUML), thereby effectively preventing enhanced permeability to intestinal substrates and pathogens.^145,146^ Moreover, butyrate enhances the assembly of tight junctions in the Caco-2 cell monolayer by activating AMP-activated protein kinases, thereby reinforcing the integrity of the intestinal barrier.^53^ T-helper 17 cells (Th17), which secrete interleukin-17 (IL-17), also play a crucial role in maintaining the integrity of the intestinal barrier.^147^ Following an imbalanced HFD-induced disturbance in the gut microbiota, the functionality of lamina propria antigen presenting cells within the small intestine becomes impaired, leading to a reduction in Th17 cell population.^148^ The compromised Th17 response contributes to an elevated level of intestinal permeability.^148^

### Metabolic endotoxemia

LPS, a pro-inflammatory compound generated by gram-negative bacteria, is an essential part of their outer membrane and significantly contributes to the development and advancement of low-grade inflammation.^57^

Dysregulation of the gut microbiome induced by HFD results in increased intestinal barrier permeability, leading to intestinal leakage and facilitating the translocation of endotoxins from the gut lumen into circulation. HFD significantly enhances the abundance of LPS-containing bacteria in the gut^57^ and elevates plasma LPS levels.^149^ Moreover, culturing wild-type mouse feces in a high-fat medium promotes greater production of LPS compared to a low-fat medium.^27^ In line with these findings, Jeong et al. showed that HFD leads to elevated levels of endotoxins in both plasma and feces, while also fostering the growth of Enterobacteriaceae and enhancing in vitro endotoxin production.^17^ Supplementation with *Lactobacillus plantarum* LC27 and *Bifidobacterium longum* LC67 leads to a decrease in the populations of *Firmicutes* and *Proteobacteria* within the gut microbiota driven by HFD. Additionally, it results in reduced fecal LPS production.^150^ These findings indicate that HFD might foster the growth of gram-negative bacteria in the gut, leading to increased LPS production. This effect can potentially be mitigated by the use of probiotics. The elevated intestinal permeability and endotoxin production associated with HFD contribute to metabolic endotoxemia characterized by increased plasma endotoxin concentrations.^151^ Notably, LPS exerts various detrimental effects on intestinal function including promotion of intestinal inflammation through specific signaling pathways. The indirect effects mediated by LPS primarily involve TLR4-CD14-dependent pro-inflammatory responses.^152^ Increased plasma levels of LPS, acting through TLR4, stimulate the release of TNF-α, IL-1, and IL-6. Therefore, it is reasonable to propose that changes in gut microbiota induced by HFD may significantly contribute to the development of low-grade inflammation.^27,153^ Studies have shown that bacteria originating from the gut can be identified in blood and white adipose tissue within just one week of HFD treatment. Furthermore, this observation was less pronounced in mice deficient in microbial pattern recognition receptors such as Nod1 or CD14, indicating a common mechanism underlying both metabolic endotoxemia and metabolic bacteremia.^154^ In summary, HFD disrupts intestinal barrier function and alters microbiome composition leading to endotoxemia and bacteremia.

### Cancer

Recently conducted studies have provided compelling evidence supporting the pivotal role of gut microbiota in the development of dietary fat-associated cancers. In a study involving genetically susceptible K-ras^G12Dint^ mice, it was observed that HFD expedited the progression of intestinal tumors by inducing alterations in the composition of gut microbiota.^155^ Furthermore, when fecal samples from HFD mice with intestinal tumors were transferred to healthy K-ras^G12Dint^ mice, augmented tumorigenicity was observed. However, this effect was effectively blocked by antibiotic treatment, thereby strongly suggesting the involvement of microbiome in disease progression. Importantly, these effects were found to be independent of obesity since K-ras^G12Dint^ mice exhibited resistance to HFD-induced obesity.^155^ Similarly, HFD-induced obesity led to modifications in gut microbiome composition which subsequently resulted in increased levels of deoxycholic acid. Deoxycholic acid, a metabolite produced by gut bacteria, is known for its ability to cause DNA damage. The enterohepatic circulation of deoxycholic acid led to increased hepatic release of various inflammatory and tumor-promoting factors, thereby accelerating the development of chemically induced hepatocellular carcinoma in mice.^156^ Furthermore, the gut microbiome also plays a pivotal role in the initiation and progression of colorectal cancer (CRC). By conducting comprehensive multi-omics studies on rodents, extensive alterations were observed in the cecal bile acid metabolic profile of mice fed HFD. Notably, there was an increase in non-classical amino acid coupling of bile acid-cholic acid (AA-CA) with HFD intake. The *Ileibacterium valens* and *Ruminococcus gnavus*, possessing AA-Cas synthesis capabilities, can modulate CA signaling through FXR and TGR5 pathways, thereby enhancing Wnt signaling and facilitating the proliferation of intestinal stem cells. This process plays a crucial role in the initiation and progression of CRC.^157^

### Central nervous system disorders

The gut microbiota communicates with the central nervous system through the brain-gut axis, exerting influence on brain function and behavior. Germ-free mice exhibited heightened motor activity and reduced anxiety-related behaviors.^158^ A mouse model of autism displayed significant changes in microbial composition, which were ameliorated by treatment with susceptible Bacteroides symbiosis found in humans, leading to improvements in communication, anxiety levels, and sensorimotor deficits.^159^ Furthermore, a study involving patients with Parkinson’s disease revealed alterations in the gut microbiome of affected individuals.^160^ While it is evident that the gut microbiota impacts central nervous system disorders, there remains limited research on HFD-induced changes within this process. However, transplantation of HFD-associated gut microbiota into mice has been reported to induce selective disruptions affecting exploration behavior, cognition, stereotypical behavior as well as potential associations with increased neuritis and impairment of cerebrovascular homeostasis.^161^ HFD affects gut microbes, causing them to release a large amount of leucine, which activates the mTORC1 signaling pathway in myeloid progenitor cells. This promotes the production and differentiation of myeloid-derived inhibitory cells (PMN-MDSCs) with polymorphonuclear morphology, further advancing cancer progression.^102^

## Targeting the gut microbiota to treat HFD-induced intestinal diseases

Recent research has explored the potential of targeting the gut microbiota as a therapeutic approach.^162^ Although still in its early stages, several studies have reported promising results. Currently, most investigations have utilized probiotics to address HFD-induced dysbiosis. Probiotics are live microorganisms that, when ingested in sufficient quantities, confer health benefits to the host by aiding digestion and nutrient absorption, maintaining gastrointestinal homeostasis, and improving key metabolic disease risk factors such as body mass index, fasting blood glucose levels, alanine and aspartate aminotransferase.^163,164^ Treating mice fed HFD with *Bifidobacterium animalis ssp. lactis* 420 (B420) can lead to reductions in fat mass, improvements in glucose tolerance, decreases in LPS levels, and reductions in inflammation.^165^ In line with these results, clinical studies investigating the supplementation of B420 have demonstrated its ability to significantly reduce body fat mass, waist circumference, and trunk fat accumulation, particularly in the central region. Additionally, B420 may decrease energy intake by modulating satiety hormones such as PYY. The effects were more pronounced when B420 was combined with prebiotics like polydextrose (PDX), indicating that B420 has potential benefits for metabolic health.^166^ Similarly, *Saccharomyces boulardii* has been shown to decrease body weight, fat mass, liver steatosis, and inflammatory response in *db/db* micemice by modulating intestinal microbial composition.^167^ Additionally, the study demonstrated that HFD mice treated with *Lactobacillus curvatus* HY7601 and *Lactobacillus plantarum* KY1032 experienced less weight gain and fat accumulation while also exhibiting reduced plasma insulin levels, leptin levels, total cholesterol levels, and hepatotoxic biomarkers associated with changes in gut bacterial composition and diversity.^168^ A randomized controlled trial conducted among Greek patients with T2D demonstrated that daily supplementation with multi-strain probiotics LactoLevureR (comprising *Lactobacillus acidophilus, Lactobacillus plantarum, Bifidobacterium lactis*, and *Saccharomyces boulardii*) for 6 months significantly enhanced blood glucose control, reduced total cholesterol levels, and decreased waist circumference. Additionally, it positively influenced the composition of gut microbiota, including specific genera, metabolites, and key enzymes associated with diabetes.^169^ In healthy adults and children, the gut microbiota usually comprises 1%-4% of the prebiotic bacterium *Akkermansia muciniphila*.^170^ *Akk. muciniphila* possesses a unique survival advantage due to its ability to utilize mucin. Its distinctive structure enables *Akk. muciniphila* to regulate intestinal barrier integrity and enhance intestinal permeability in HFD mice.^171^ Moreover, the Type IV pili of *Akk. muciniphila* is capable of directly interacting with host immune receptors, regulating the expression of genes associated with hepatic lipid synthesis and inflammatory responses, and preserving balance within the intestinal immune system.^172–175^ Additionally, *Akk. muciniphila* has the ability to secrete oligosaccharides and short-chain fatty acids that promote microbiome enrichment and reduce the risk of obesity.^176,177^ Furthermore, plant-derived compounds have the potential to positively influence gut microbiota composition in obesity studies. Extracts from cranberries, which are rich in polyphenols, have been shown to safeguard mice against diet-induced obesity and metabolic disturbances by boosting the abundance of *Akkermansia* species.^178^ Prolonged supplementation with *Akk. muciniphila* can enhance the mucus layer thickness in the intestinal barrier and simultaneously reduce the expression of genes linked to inflammation.^179^ Despite the limited data from current human trials, the successful use of intestinal bacterial transplantation in treating *Clostridium difficile* infections provides a new method for preventing and managing chronic conditions linked to high-fat diets in humans. In conclusion, the use of probiotics and prebiotics holds great promise in alleviating metabolic disorders caused by HFD, reducing fat accumulation, inflammatory response, and insulin resistance, while enhancing intestinal barrier function. This approach offers a promising new direction for the prevention and management of HFD-associated chronic. To more accurately and effectively modify the microorganisms in the host gut, genetic engineering techniques have recently been employed to alter specific microorganisms, endowing them with targeted functions for the treatment and prevention of chronic diseases.^180–183^ Engineered probiotics have also been utilized in HFD-induced chronic diseases. N-acylphosphatidylethanolamines (NAPEs) are lipid derivatives involved in anorexia signaling molecules in the small intestine. When mice on HFD were administered with engineered bacteria *EcN* expressing NAPEs, significant inhibition of weight gain was observed in the obese mouse model. Moreover, this intervention reduced liver inflammation and alleviated early symptoms of fibrosis.^184,185^ Additionally, modification of *Lactobacillus* to secrete GLP-1 and induce insulin secretion has shown improvement in hyperglycemia symptoms in diabetic rat models.^186^ Tissue accumulation of Pyrroloquinoline quinone (PQQ) can prevent oxidative damage both locally within the liver and systemically throughout the body. Combined with SCFAs, PQQ can effectively reduce hyperlipidemia. Chaudhari et al. constructed engineered *EcN* capable of expressing mtlK and fdh enzymes, resulting in increased SCFAs and PQQ levels along with decreased body weight and blood glucose concentration in mice.^187^ The engineered bacterium BsS-RS06551, which produces butyric acid, was developed using *Bacillus subtilis* as the cellular chassis. Administration of BsS-RS06551 significantly attenuated obesity induced by HFD and exhibited favorable effects on host glucose and lipid metabolism as well as gut microbial composition.^188^

## Conclusions and future perspectives

Long-term consumption of HFD is associated with an increased risk of obesity, diabetes, liver metabolic disease, cardiovascular disease, chronic intestinal inflammation, cancer, and other chronic diseases. The gut microecological disorders caused by dietary fats may play a more pivotal role in the promotion of these chronic diseases than genetic predispositions. Mechanistic studies have identified specific gut bacterial taxa that are linked to the disease risks associated with HFD. A predominant finding across most studies is that an elevated *Firmicutes*/*Bacteroidetes* ratio, along with alterations in microbial communities at the family, genus, and species levels, represents a key characteristic of HFD-induced shifts in the gut microbiota. However, the pathogenesis of HFD-related chronic diseases is likely the result of an intricate. There is also an interaction between chronic diseases directly driven by fat accumulation and gut microbiota making it difficult to determine the causal relationship between microecological dysregulation and morbidity. Therefore, HFD-driven specific intestinal bacterial type disorders and HFD-related chronic diseases need to be studied as partially independent yet closely related mechanisms. Published findings suggest that maintaining a balanced diet with appropriate fat content is crucial not only for host health, but also for the gut microbiome. The dysbiosis of intestinal microbes plays a significant role in the development of chronic diseases associated with HFD. The prevailing perspective posits that HFD induces alterations in specific intestinal microbiota, leading to changes in microbial metabolic reactions within the intestine. This results in an increased production of disease-promoting metabolites or disruption of synthesis pathways for beneficial metabolites that can be positively regulated. Consequently, this impairs the host’s glucose and fat metabolism, compromises intestinal barrier function, disrupts signal transduction processes, and promotes endotoxin accumulation.

Based on our literature discussion regarding the impact of HFD on gut microbiome alterations, the investigation of specific microbial metabolites predominantly relies on *in vivo* studies conducted in target tissues or cells, often supplemented by rodent-based experimental models. Although mouse studies have provided valuable insights into the role of gut microbes and their metabolites in HFD-induced chronic metabolic diseases, translating these findings to human applications faces significant challenges. Firstly, species differences limit direct extrapolation, as mice and humans differ substantially in gut microbiome composition, metabolic pathways, and genetic backgrounds. Key microbiota and metabolites in humans may be absent or less abundant in mice, and their mechanisms of action can vary markedly. Secondly, experimental conditions in mouse studies often fail to replicate real-world human lifestyles. The composition of high-fat diets used in mice differs from typical human diets, and controlled laboratory environments cannot capture the complexity of human factors such as stress, sleep, and medication use. Additionally, mouse studies predominantly use inbred strains with homogeneous genetics, whereas humans exhibit significant genetic and microbiome diversity, leading to variability in microbial-host interactions. Human chronic metabolic diseases arise from a complex interplay of genetic, dietary, lifestyle, and environmental factors, which mouse models cannot fully replicate. Mouse studies also typically involve short-term interventions, which may not reflect the long-term progression of human diseases, and dosages or exposure durations may not align with real-world scenarios. Ethical and practical constraints further limit human studies, making long-term dietary interventions challenging to implement. Moreover, small sample sizes in mouse experiments can introduce biases when extrapolating results to human populations. To address these limitations, future research should prioritize rigorously designed, long-term human clinical studies, including cohort studies and intervention trials, with clearly defined endpoints and large sample sizes. Multi-omics technologies (e.g., genomics, metabolomics) should be employed to comprehensively analyze microbiome-host interactions. Cross-species validation using multiple animal models and interdisciplinary collaboration will enhance the reliability and translational potential of findings. In summary, while mouse studies provide valuable insights, greater emphasis on human research, technological innovation, and cross-disciplinary collaboration is essential for translating these findings into clinical applications.

The existing literature on targeting the microbiota as a therapeutic approach for diet-induced chronic diseases is promising and suggests potential as a complementary strategy to modulate gut microbiota composition. However, a comprehensive understanding of viable pharmacological agents in this context remains insufficient, and critical aspects such as optimal dosage, timing, and administration frequency have yet to be determined. Future research should prioritize the following directions to elucidate the role of gut microbes and their metabolites in high-fat diet-induced metabolic diseases and facilitate clinical translation. Leveraging the significant individual variability in gut microbiome composition and function, future studies should integrate multi-omics and AI-driven approaches to develop precise dietary protocols tailored to individual microbiome profiles. For example, diets enriched with prebiotics, probiotics, or specific microbial metabolites could be designed to target specific microbial communities, enhancing metabolic health. Additionally, exploring variations in dietary responses across diverse populations (e.g., age, gender, ethnicity) will refine personalized nutrition strategies. Advances in synthetic biology and gene editing enable targeted microbiome modifications to modulate host metabolism and improve disease phenotypes. However, the safety, stability, and long-term effects of these interventions in complex gut environments require rigorous validation. Translating basic research into clinical practice remains challenging. Key priorities include developing precision microbiome-based therapies, such as FMT, probiotic/prebiotic combinations, and metabolic pathway-targeted interventions. Large-scale clinical trials are essential to evaluate the long-term safety and efficacy of these interventions in preventing, treating, and reversing metabolic diseases. Furthermore, exploring synergies between microbiome-based therapies and other treatments (e.g., pharmacological, lifestyle, or surgical interventions) will optimize outcomes and improve patient adherence.
